# Kaurenic acid: An *in vivo* experimental study of its anti-inflammatory and antipyretic effects

**DOI:** 10.4103/0253-7613.70205

**Published:** 2010-10

**Authors:** Miriam C. Sosa-Sequera, Omar Suárez, Nelson L. Daló

**Affiliations:** Research Unit in Experimental Pharmacology, School of Medicine, Universidad Centroccidental Lisandro Alvarado, Barquisimeto, Venezuela

**Keywords:** Antipyretic, anti-inflammatory, kaurenic acid, tetracyclic diterpenoid

## Abstract

**Objective:**

This study was designed to investigate the anti-inflammatory and antipyretic effects of kaurenic acid (KA), a tetracyclic diterpenoid carboxylic acid, using *in vivo* experimental animal models.

**Material and Methods:**

The anti-inflammatory activity of KA was evaluated in rats, using egg albumin-induced paw edema (acute test) and Freund’s complete adjuvant-induced paw edema (subacute test), whereas the antipyretic effect was studied in rabbits by peptone-induced pyresis. Acute and subacute toxicity of KA were analyzed in NMRI mice.

**Results:**

KA showed anti-inflammatory and antipyretic properties, and the effect caused was significantly dose-related (*P* < 0.001) in both cases. The mean lethal doses of KA were 439.2 and 344.6 mg/kg for acute and subacute toxicity, respectively.

**Conclusion:**

On the basis of these findings, it may be inferred that KA has an anti-inflammatory and antipyretic potential.

## Introduction

Kaurenic acid [(–)-kaur-16-en-19-oic acid] (KA) is a diterpenoid acid derived from an ent-kaurene tetracyclique hydrocarbure isolated from the aerial parts of the species *Espeletia semiglobulata*.[1] It is described that Kaur-16-en-19-oic acid derivatives may be promising compounds for leading new chemopreventive strategies as well as having contributed to development of antibiotic and antimicrobial agents.[2,3]

Ent-kaurenes and many natural derivatives of these diterpenes have significant anti-inflammatory, anti-hypertensive, and diuretic biological effects *in vivo*, as well as antimicrobial, smooth muscle relaxant, and cytotoxic actions *in vitro*.[4] Numerous findings indicate that ent-kauranes are potential anti-inflammatory agents, with a specific mechanism in which both the inhibition of nuclear factor kappa B (NF-kappa B) translocation and the consequent decrease of proinflammatory mediators are implicated. The antipyretic effects also have been analyzed in different diterpene-types.[5]

KA is a bioactive compound with demonstrated anticonvulsant, sedative,[6] and hypotensive effects.[7] This study has been carried out in order to evaluate the anti-inflammatory and antipyretic properties of KA using *in vivo* experimental animal models for inflammation and pyrexia.

## Materials and Methods

### Drugs and reagents

KA was isolated from *E. semiglobulata*, and purified as previously described.[1] KA, as sodium kaurenate [C_20_H_29_NaO_2_], a gift from Dr. Alfredo Usubillaga (Research Institute, ULA, Mérida, Venezuela) was diluted in distilled–deionised water for assays.[8,9] Only high purity reagents were used in the different assays. Acetylsalicylic acid (ASA) and Freund’s complete adjuvant (FCA) were purchased from Sigma (St. Louis, MO). Egg albumin was obtained from Merck AG (Darmstadt, Germany). Dexamethasone (DX, Decobel^®^) was from Productos Ronava (Caracas, Venezuela). Acetaminophen (ACTP) (Tempra^®^) was from Mead Johnson (Venezuela), and Peptone (PT) was from Himedia Laboratories (Mumbai, India).

### Animals

Adult male and female animals were used in this study. Sprague-Dawley rats (body weight, 240–300 g), NMRI mice (body weight, 25–35 g), and New Zealand male rabbits (body weight, 2–4 kg) were supplied by the Veterinary School of Universidad Centroccidental Lisandro Alvarado, Venezuela. All animals had free access to food and water and were kept on 12/12 h light–dark cycle. Before each study, animals were submitted to fasting for at least 12 h. Animals were managed according to the Guide to the Care and Use of Experimental Animals, Canadian Council on Animal Care (1984), and the Guide to the Use of Laboratory Animal, National Institute of Health, Bethesda, USA. (Publication 86–23, 1985).

### Evaluation of Anti-inflammatory activity

### Acute anti-inflammatory activity

This study was performed using the egg albumin model.[10] Sprague-Dawley rats (*n* = 10) were randomly allocated in three groups: an experimental group received KA (20, 40, 80, and 160 mg/kg i.p.), a standard treatment group was treated with acetylsalicylic acid (ASA: 50, 100, 200, and 250 mg/kg), and a control group received 0.9% saline solution (1 mL). In order to induce inflammation, all animals were injected subcutaneously (s.c.) in the left hind paw with 0.1 mL of an albumin solution diluted as 0.5% w/v in 0.9% saline solution, 30-min post-treatment. Acute inflammation was measured as the linear diameter using callipers. Measurements were carried out every 30 min for 5 h after administration of experimental and control treatments.[11]

The anti-inflammatory effect of KA and ASA was calculated and compared with the inflammation in the control group. Acute anti-inflammatory activity was also confirmed by the volume displacement method[12] using a Digital Plethysmometer LE 7500 (Letica, Barcelona, Spain). The mean effective dose (ED_50_) was calculated for KA and ASA, and the therapeutic index was determined in relation to acute LD_50_.

### Subacute anti-inflammatory activity

These experiments were performed using the Freund’s adjuvant arthritis model. The administration of the Freund’s complete adjuvant (FCA, 0.1 mL, s.c.) was done according to what was previously described.[13] KA (20, 40, 80, and 160 mg/kg/day) was administered during 3 days. Dexamethasone (DX: 0.2, 2.0, and 3.8 mg/kg/day, i.p.) and saline 0.9% (1 ml/day, i.p.) were given by the same route at the same periods of time and were used as control groups. The subacute inflammation level was measured by the volumetric method using plethysmometer and by determining the linear diameter in a parallel assay.[11,12] Paw swelling was determined at 0, 1, 6, 24 h, and during the next 6 days. The average volume and thickness of the paw in the experimental group (KA) was calculated and compared with values obtained from the standard and control groups (DX and saline) according to what was described before.[13] The ED_50_ was calculated, and the therapeutic index was estimated in relation to the values of subacute LD_50_.

### Acute antipyretic activity

One hour prior to the assay, the animals were acclimatized at room temperature (21°C). New Zealand rabbits (*n* = 6) were randomly divided into four groups. Three of them were treated with peptone (PT), a pyrexia inductor, in 1% solution at doses of 0.1 mL/kg, i.v. and control group received only 0.9% saline (5 mL, i.v.). Experimental group treated with PT, also received KA in a doses of 10, 20, 40, 80, and 160 mg/kg i.m. The standard group received acetaminophen (ACTP: 25, 50, and 100 mg/doses, i.m.). PT was given to all groups after recording basal body temperature for 1 h poststabilization in the cuff. Core body temperature (*T*_b_) was measured in the rectum every 15 min during 4 h as described elsewhere.[14] Pyretic or antipyretic effects were considered when the differences between maximal and minimal temperatures were 0.6°C or larger. The ED_50_was calculated for experimental and control groups, and the therapeutic index was determined with respect to acute LD_50_.

### Acute and subacute toxicity testing

NMRI mice (*n* = 10) received KA intraperitoneally. In acute assays, the animals were treated with a single-dose ranging between 10 and 600 mg/kg body weight and mice were observed for gross behavioural effects and mortality for a period of 24 h. The control group received saline solution (0.5 mL). Accordingly, in subacute studies, the selected doses of KA were 40, 80, 160, 320, and 460 mg/kg, i.p. administered during 5 days. The LD_50_ was estimated.

### Statistical analysis

The percentage of lethality was determined using nonlinear curve regression analysis. Variance analysis (two-way ANOVA) followed by a Bonferroni post-test were used for significance analysis of differences of inflammation and pyresis data, respectively. The data were expressed as mean ± SD obtained from at least three independent experiments performed by triplicate. Values *P* < 0.05 were reported as statistically significant. All statistical analysis were done using Prism Software version 4.0 (GraphPad Software Inc., San Diego, CA).

## Results

### Acute anti-inflammatory activity

**Figure 1:**
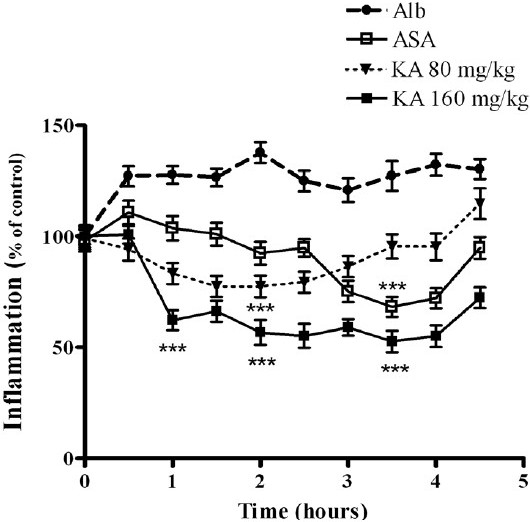
Effect of KA on albumin-induced acute edema. Data were calculated as percent of control and expressed as mean ± SD (n = 10) of triplicate measurements of three separated experiments. ***P < 0.001 vs. edema-inductor.

KA showed dose-related anti-inflammatory effects at 2 h with an ED_50_ of 83.37 ± 0.29 mg/kg (95% CI: 3.51–197.8) which was comparable to ASA = 97.62 ± 0.10 mg/kg at 2 h) (95% CI: 36.50–261.1). The anti-inflammatory effect was 60.26% and 81% for doses of 80 and 160 mg/kg, respectively. These results were statistically significant when compared to those of control group receiving albumin plus saline (*P* < 0.01; *P* < 0.001). By comparison, ASA 200 mg/kg inhibited the edema significantly (*P* < 0.01) which represented 58.93% of anti-inflammatory activity at 3.5 h. Similarly, plethysmometric measurements showed 77.55% inhibition for KA (160 mg/kg i.p.) that was comparable to ASA (60.45%, *P* < 0.001). Anti-inflammatory activity of KA compared to control groups is shown in Figure 1. The therapeutic index value corresponded to 5.26.

### Subacute anti-inflammatory activity

**Figure 2:**
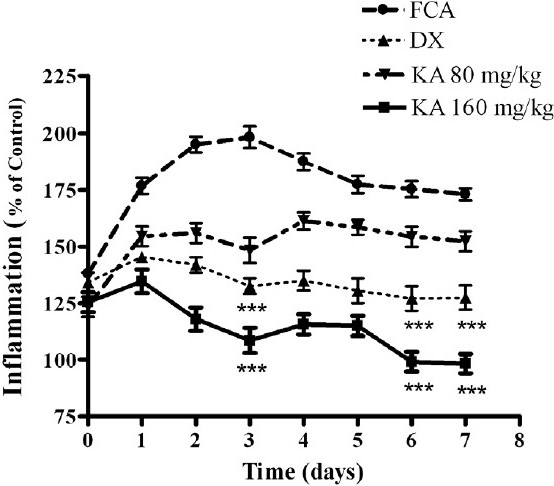
Effect of KA on adjuvant-induced subacute edema. Data were calculated as percent of control and expressed as mean ± SD (n = 10) of triplicate measurements of three separated experiments. ***P < 0.001 vs. edema-inductor.

Evaluation of the subacute anti-inflammatory effect using the two methods indicated that KA caused inhibition from 50% and 89.57% at doses of 80 and 160 mg/kg, respectively, after 2 days of treatment (*P* <0.001). The anti-inflammatory effect was dose-related. The ED_50_ was found to be 74.85 ± 0.50 mg/kg (95% CI: 62.44–89.74) in relation to DX = 1.41 ± 0.51 mg/kg/day (95% CI: 0.04–46.79) at 3 days post-treatment, being the therapeutic index for KA of 4.60 in this subacute model. The percent inhibition for DX (2 mg/kg) was 66%, and it was significantly different from FCA plus saline control animals (*P* < 0.001) [Figure 2]

### Acute antipyretic activity

**Figure 3:**
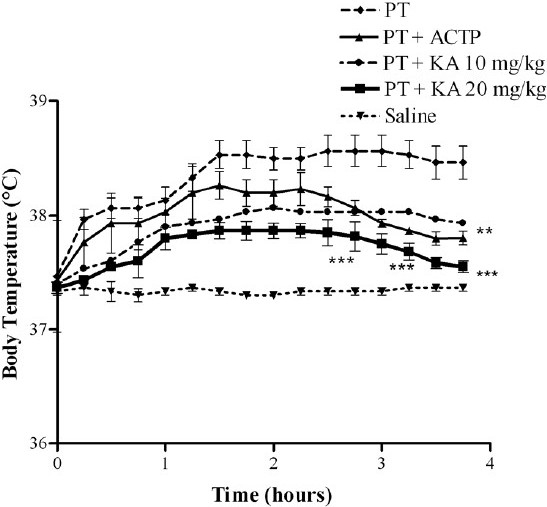
Effect of KA on body temperature in conditions of peptone-induced fever. Results are expressed as mean ± SD (n = 6) of triplicate measurements of three separated experiments. **P < 0.01, ***P < 0.001 vs. increment in PT-induced fever.

The measurements of body temperature (*T*_b_) are represented in Figure 3. The saline group showed a constant basal *T*_b_ of 37.6 ± 0.15°C over a 4-h period. An increase in *T*_b_ to 38.56 ± 0.18°C was observed at 1 h post-PT. However, when animals were treated with KA (10 and 20 mg/kg) at 2.30 h after PT injection a decrease of 0.6 ± 0.2°C and 1.2 ± 0.4°C, respectively, was found. This finding was significantly different in animals treated with PT only (*P* < 0.01 and *P* < 0.001, respectively) and mimicked with ACTP, which showed a decrease of 0.81 ± 0.43°C (*P* < 0.01). The effects were dose-dependent. The ED_50_ for the antipyretic effect of KA was 23.56 ± 0.9 mg/kg (95% CI: 21.36–25.99) in relation to 44.38 ± 0.41 mg/dose (95% CI: 1.37–143) for ACTP. The therapeutic index of KA for acute model was 18.64.

### Acute and subacute toxicity

The estimated LD_50_ for KA in the acute assay was 439.2 mg/kg, i.p. (95% CI: 413.2–466.9). In contrast, LD_50_ in the subacute study performed during 5 days was found to be 344.6 mg/kg, i.p. (95% CI: 243.1–488.4). During treatment, mice showed some degree of somnolence, weakness of the hind limbs, and accelerated breathing at dose of 10 mg/kg and higher.

## Discussion

The results of the study suggest that KA has anti-inflammatory and antipyretic activities in the experimental models studied. This indicates that KA is a bioactive diterpenoid; which corresponds with the reports that describe the properties of diterpenes kaurenoic.[8,15–18] KA was also as potent as ASA and ACTP in the acute models, but with minor potency in relation to DX. The ED_50_ value for the anti-inflammatory effect represented 1/6 of LD_50_, whereas the ED_50_ value for the antipyretic effect was 1/20 of LD_50_. Although the therapeutic index for the anti-inflammatory activity was low, no deaths were observed in these animals at the doses used in the assays.

The development of acute edema in the paw of the rat has been described as a three-phase event. It is mainly attributed to the release of histamine, serotonin in the initial phase (0–2 h); cytokines, in the second phase (3 h); and prostaglandin, in the third phase (>4 h).[19] The anti-inflammatory effects of KA observed in the acute model lasted for up to 5 h. This shows that KA acted on all inflammatory phases, whereas ASA acted much slowly. In addition, KA in the subacute model maintained its anti-inflammatory effect for 6 days, which was more accentuated in relation to DX; nevertheless, higher doses were required. These findings suggest that KA exhibited a potent capacity for inhibiting acute and subacute edema.

Although elucidating the mechanism of action of KA was not the main aim of this study; the anti-inflammatory effect of most terpenes has been explained in terms of the inhibition of NF-kappa B. In this regard, it has been reported that ent-kaurene diterpenes inhibit the expression of inducible nitric oxide synthase (iNOS) and the release of tumor necrosis factor-alpha (TNF-α).[20] It is also known that several diterpenoids isolated from the Chinese herb *Isodon rubescens* are potent inhibitors of NF-kappa B transcriptional activity which subsequently target iNOS and cycloxygenase-2 (COX-2), with the latter being less sensitive to diterpenoids.[21] Other diterpenoids isolated from Chinese herbal remedy (*Tripterygium wilfordii* Hook.f.) inhibit the interleukin-1α-induced production of PGE2 by selectively suppressing the gene expression and production of COX-2, but not those of COX-1.[22] Furthermore, recently the ent-kaurene isolated from the roots of *Siegesbeckia pubescens* was shown to inhibit iNOS, COX-2, and TNF-α protein via down-regulation of NF-kappa B binding activity.[23] In relation to this mechanism, we have previously reported evidence that KA, administered in mice (1 mg/kg), inhibits the expression of genes for the proteins iNOS, eNOS, neuronal NOS (nNOS), and Bcl-xL in murine melanoma cells.[24] In addition, it appears that the effect of terpenes on the gene expression of COX-2 is less sensitive than that of the iNOS gene.[23] Therefore, kaurenes could be acting as anti-inflammatory agents through a novel mechanism of action totally different from that of common nonsteroidal anti-inflammatory drugs (NSAID) that inhibit COX-1 with subsequent side effects on the gastric mucosa and platelets aggregation. The results obtained in our study are coincident with these reports in relation to the potent anti-inflammatory effect of diterpenes.

The antipyretic effect of diterpenes could also be explained by its potent inhibition of iNOS, because NO has been shown to be an important mediator of febrile responses. In this regard, when fever is induced, activation of iNOS is responsible for triggering and maintaining lipopolysaccharide-induced fever in rats.[25,26] In agreement with these data, KA-induced antipyretic effect with a latency of 1 h, during a period of 4 h and with a good therapeutic index. As an antipyretic, KA could be working via a nitrergic mechanism due to its inhibition of NOS;[27] but a prostanoid pathway has not been investigated yet.

In this work, evidence is presented that KA has anti-inflammatory and antipyretic effects; nonetheless, further studies are needed for evaluating levels of NO or interleukins to explain underlying mechanisms involved in these events. The discovery of diterpenes as specific inhibitors capable of blocking NF-kappa B activation could be an excellent approach for the treatment of inflammatory diseases. Our results justify further investigations in order to evaluate diterpenes as potentially useful agents for the treatment of inflammation and fever.
